# Enhancing Quality of Life and Emotional Well‐Being in Atopic Dermatitis Patients: Clinical Evidence of a Dermocosmetic Lipid‐Replenishing Regimen

**DOI:** 10.1111/jocd.70658

**Published:** 2026-01-14

**Authors:** Marie Gaudin, Aurélie Geoffroy

**Affiliations:** ^1^ Laboratoires Nigy (Topicrem) Rueil Malmaison France

**Keywords:** atopic dermatitis, lipid‐replenishing regimen, pruritus relief, quality of life, SCORAD, skin barrier function, well‐being

## Abstract

**Background:**

Atopic dermatitis (AD) is a chronic inflammatory skin disorder characterized by impaired skin barrier function, xerosis, and pruritus, significantly affecting quality of life (QoL). Effective dermocosmetic strategies are needed to alleviate symptoms and improve well‐being.

**Aims:**

This study assessed the efficacy of a lipid‐replenishing dermocosmetic regimen, including a cleansing oil and emollient balm (Topicrem, Laboratoires NIGY, France), in adults with moderate to severe AD.

**Patients/Methods:**

A 4‐week clinical study was conducted under dermatological supervision on 21 adults with moderate to severe AD. Patients applied the DA PROTECT Replenishing Cleansing Oil and Emollient Balm (Topicrem) once or twice daily. Efficacy was evaluated via TransEpidermal Water Loss (TEWL), corneometry, SCORAD (SCORing Atopic Dermatitis) index, and patient‐reported outcomes. The impact on QoL was assessed using the Dermatology Life Quality Index (DLQI).

**Results:**

After 4 weeks, TEWL decreased by 27%, and skin hydration increased by 89% indicating a reinforced skin barrier and improved moisturization. The SCORAD index improved by 27%, pruritus decreased by 61%, and sleep quality improved by 62%, reflecting reduced disease severity. The DLQI score improved by 68%, demonstrating a strong correlation between the dermocosmetic regimen and enhanced QoL, particularly in items related to symptoms, sleep, social life, and emotional well‐being. Patient satisfaction was high, with 81% rating efficacy as “good to very good” and 90% approving cosmetic acceptability.

**Conclusion:**

The investigational lipid‐replenishing regimen significantly improved skin barrier function, hydration, pruritus relief, and QoL in patients with moderate to severe AD. These findings highlight the importance of dermocosmetic interventions in AD management, reducing discomfort, and enhancing patient well‐being.

## 1 Introduction

Atopic dermatitis (AD) is more than a chronic inflammatory skin disease. It is a daily burden that goes beyond the visible manifestations of xerosis, erythema, and intense pruritus. Affecting approximately 10% of adults and up to 20% of children worldwide [1], AD results from a multifactorial interplay between genetic predispositions, epidermal barrier dysfunction, immune dysregulation, and environmental influences [2]. Despite well‐documented clinical features, the psychosocial dimension of AD remains insufficiently addressed. The persistent itch‐scratch cycle, sleep disturbances, and social embarrassment contribute to a significant decline in quality of life (QoL), often comparable to that observed in chronic conditions such as diabetes or depression [3]. Recent insights from the Global Report on Atopic Dermatitis further underscore the systemic impact of the disease, highlighting its effects on psychological well‐being, school and work productivity, and social relationships [1].

Importantly, impaired skin barrier function is not only a hallmark of AD, but also a central factor contributing to disease flare‐ups, increased transepidermal water loss, and susceptibility to irritants and allergens penetration and microbial colonization [4]. This underscores the importance of skin barrier maintenance as a foundational component of both acute and long‐term management strategies. While pharmacological treatments remain indispensable for controlling inflammation and immune responses, they may not sufficiently address the chronic biophysical fragility of the skin barrier, a core issue in AD management. Furthermore, the prolonged use of corticosteroids raises concerns regarding side effects, patient adherence, and the emergence of “corticophobia.” In this context, dermocosmetic care represents a complementary approach that targets the restoration of skin barrier integrity, supports symptom relief, and contributes to overall patient well‐being [5].

Despite growing consensus on the benefits of emollient use in AD, few studies have evaluated the additive value of a complete dermocosmetic regimen encompassing both cleansing and moisturizing phases. Yet, gentle daily hygiene, when formulated to preserve lipid content and barrier function, may enhance therapeutic efficacy and promote long‐term adherence. A lipid‐replenishing regimen that combines gentle active cleansing with intensive moisturization has the potential to address both the physical and emotional dimensions of the disease, particularly in moderate to severe cases.

This study under dermatological control investigates the clinical performance and patient‐reported outcomes of a dermocosmetic regimen combining a lipid‐replenishing cleansing oil and emollient balm, specifically designed for atopic‐prone skin. By integrating objective dermatological parameters with measures of well‐being and QoL, this work aims to clarify the broader role of comprehensive skincare in the multidimensional management of AD.

## 2 Material and Methods

### 2.1 Study Design

An open‐use test was carried out in a dermatological center in South Africa to evaluate the benefit of a lipid‐replenishing skincare regimen, under routine home‐use conditions; both the cleansing oil and the emollient balm were applied once or twice daily on the entire body, over a 4‐week period, on 21 subjects with moderate to severe AD.

The aim was to assess improvements in clinical severity, skin barrier function, skin hydration, and QoL. Evaluations were performed at baseline (D0) and after 4 weeks (D28). Clinical scoring of AD was assessed by the dermatologist investigator and through self‐assessment using the SCORAD index.

### 2.2 Study Participants

This study enrolled 21 volunteers (29% male, 71% female), aged between 18 and 70 years, with Fitzpatrick skin phototypes I to V, and diagnosed with moderate to severe AD. Eligibility was confirmed through a standardized questionnaire and clinical examination.

*Inclusion criteria* required participants to be adults (18–70 years), male or female, with skin phototypes I to VI, presenting with AD lesions on the body and a SCORAD index ≥ 25 at baseline. Skin type and self‐reported sensitivity were not restrictive factors.

*Non‐inclusion criteria* comprised absence of an appropriate wash‐out period, pregnancy or breastfeeding, or unstable health conditions that could place the subject at undue risk. Subjects receiving medical treatments, vaccinations (except COVID‐19 vaccination), or presenting a history of cosmetic or drug allergies incompatible with the investigational products were excluded. Additional criteria included recent excessive sun exposure, surgical or chemical/physical procedures on the test areas, or the recent use of exfoliating, self‐tanning, cosmetic, or pharmaceutical products on the concerned areas outside of the permitted wash‐out conditions. Subjects with severe irritation or abnormalities on the test sites (other than AD) were also excluded.

### 2.3 Study Products

The study evaluated a lipid‐replenishing skincare regimen composed of:

- DA PROTECT Replenishing Cleansing Oil (TOPICREM, Laboratoires NIGY, France)—a soap‐free, ultra‐gentle cleansing oil formulated with linseed oil derivatives and camelina oil, known for their high content of omega‐3 and omega‐6 fatty acids. The formula also includes glycerin, which helps maintain skin hydration [6]. This formula was developed to cleanse the skin while reinforcing the skin barrier.
- DA PROTECT Emollient Balm (TOPICREM, Laboratoires NIGY, France)—a rich, nourishing balm, classed as an Emollient “plus” [7], containing linseed oil, shea butter, and allantoin, selected for their moisturizing and soothing properties. This formula provides intense hydration, supports barrier function restoration, and helps reduce dryness and discomfort associated with AD.

### 2.4 Assessments and Measurements

The efficacy of the skincare regimen was assessed using a combination of instrumental, clinical, and subjective methods. Instrumental evaluations focused on two key parameters reflecting skin barrier function and hydration.

TEWL was measured using a Tewameter. This non‐invasive method quantifies the amount of water lost through the stratum corneum and is commonly used to assess the integrity of the skin barrier. A decrease in TEWL values is indicative of an improvement in skin barrier function, whereas an increase would suggest a defective barrier [8].

Skin surface hydration was assessed by measuring electrical capacitance using a Corneometer. This technique is based on the principle that water has a high dielectric constant and capacitance values are directly related to the water content in the upper layers of the epidermis. An increase in capacitance is interpreted as an improvement in skin hydration [8].

Clinical severity of AD was evaluated using the SCORAD index, which ranges from 0 to 103 and incorporates both objective and subjective symptoms. The clinical assessment is based on three components: the extent of the lesions, the intensity of the symptoms: xerosis, erythema, edema/papules, oozing/crust, excoriation, lichenification, each rated on a 4‐point scale (0 = none to 3 = severe) and subjective symptoms, pruritus and sleep loss (insomnia) reported by the patient [9]. This assessment was conducted both by the dermatologist investigator and through self‐assessment by subjects, at baseline and after 4‐week, to capture both objective clinical changes and perceived symptom improvement.

In parallel, clinical assessment of skin irritation was conducted by the dermatologist investigator under controlled conditions at D0 and D28, using an investigator‐designed 10‐point visual scale (0 = no lesion, no redness, edema, papules, crust, excoriation, and not dry skin; 9 = important lesion, marked redness, edema, papules, crust, excoriation, and very dry skin), evaluating redness, edema, papules, crusting, excoriations, and dryness.

Subjects completed self‐assessments at D0 and D28. Skin comfort was rated on a 10‐point subjective scale (0 = very uncomfortable; 9 = very comfortable), and skin irritation was self‐evaluated using a structured visual analog scale (0–9), categorized as none, slight, moderate, or marked.

Subjects also completed the Dermatology Life Quality Index (DLQI) questionnaire at D0 and D28 to assess the impact of the dermatitis on QoL and well‐being. It was completed in the presence of the dermatologist investigator to ensure accurate reporting.

At D28, subjects were asked to complete a product‐specific self‐evaluation questionnaire. The questionnaire focused on perceived efficacy and cosmetic acceptability, with responses rated on a 4‐point Likert scale (“agree,” “somewhat agree,” “somewhat disagree,” “disagree”).

A global appraisal of the overall efficacy and cosmetic performance of the skincare regimen was conducted at the end of the study (D28). This qualitative assessment provided further insight into user satisfaction and perceived benefit.

### 2.5 Statistical Analysis

For instrumental measurements, mean values and standard error of the mean (SEM) were calculated at each time point.

For clinical evaluations (SCORAD) and subject‐reported outcomes, mean values and standard deviations (SD) were determined. The normality of data distribution was assessed using the Shapiro–Wilk test. In cases where the data followed a normal distribution, statistical comparisons between baseline (D0) and posttreatment values (D28) were performed using paired two‐tailed Student's *t*‐tests. When normality was not confirmed, the nonparametric Wilcoxon signed‐rank test (two‐tailed) was applied. A *p*‐value of < 0.05 was considered statistically significant, while values between 0.05 and 0.10 were interpreted as borderline significant.

Changes in DLQI scores between D0 and D28 were analyzed using paired Student's *t*‐tests, with results expressed as mean ± SD and a significance level set at *p* < 0.05.

## 3 Results

A total of 21 subjects with clinically diagnosed AD and a baseline SCORAD index ≥ 25 (mean: 42.8; range: 28.1–62.1) were included in the analysis. The study population consisted exclusively of adults (mean age: 35.2 years; range: 18–64), with a majority being female (71%). Skin type was determined by the dermatologist investigator during clinical examination. A total of 86% of participants were classified as having dry skin and 14% as having very dry skin, based on a clinical grading scale ranging from 0 (normal skin) to 3 (very dry skin) evaluating roughness, scaling, and lack of suppleness.

Skin sensitivity was reported by 90% of participants, based on self‐assessment using a standardized questionnaire addressing sensations such as stinging, burning, and discomfort in response to environmental or cosmetic factors. All participants had active AD lesions at inclusion.

In terms of phototype distribution, 10% were phototype I, 14% phototype II, 43% phototype III, 19% phototype IV, and 14% phototype V, reflecting a diverse population with predominantly mixed or darker skin tones (67% colored skin, 29% Caucasian, 5% African origin).

### 3.1 Clinical Parameters

**FIGURE 1:**
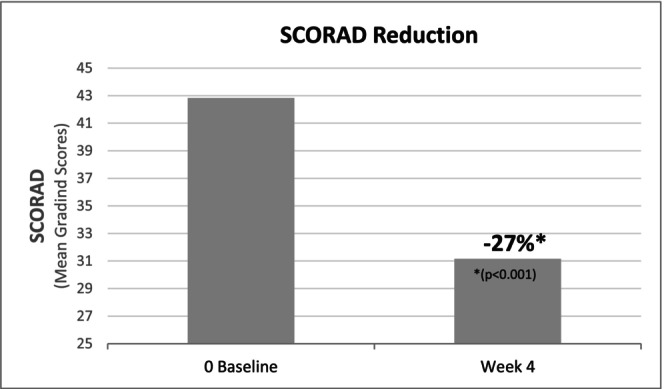
Reduction of SCORAD index after 28 days of daily application of the dermocosmetic regimen.

The SCORAD index showed a statistically significant reduction after 28 days of daily application of the two‐product regimen. The mean SCORAD decreased by 27% (from 42.83 ± 9.05 at baseline to 31.16 ± 11.67 at D28; *p* < 0.001) (Figure 1). The extent of lesions decreased significantly by 21% (from 19.93 ± 13.10 at baseline to 15.71 ± 10.75 at D28; *p* = 0.019).

The most notable clinical improvements were observed in the subjective symptom domain, with a 59% reduction in pruritus (7.50 ± 1.61 to 3.05 ± 2.29; *p* < 0.001) and a 62% reduction in insomnia (5.18 ± 2.39 to 1.97 ± 1.83; *p* < 0.001). Overall, the subjective symptom score decreased by 61% (from 12.68 ± 3.24 to 5.00 ± 3.19; *p* < 0.001).

Investigator‐assessed (visual) skin irritation on the whole body significantly decreased after 4 weeks (4.50 ± 1.00 to 3.45 ± 1.70; *p* = 0.005), corresponding to a 23% reduction. Self‐assessments by participants mirrored these findings, showing a 51% decrease in perceived body skin irritation (7.40 ± 1.31 to 3.60 ± 2.70; *p* < 0.001), along with a significant 198% improvement in perceived skin comfort (2.25 ± 1.55 to 6.70 ± 2.32; *p* < 0.001).

### 3.2 Instrumental Parameters

**FIGURE 2:**
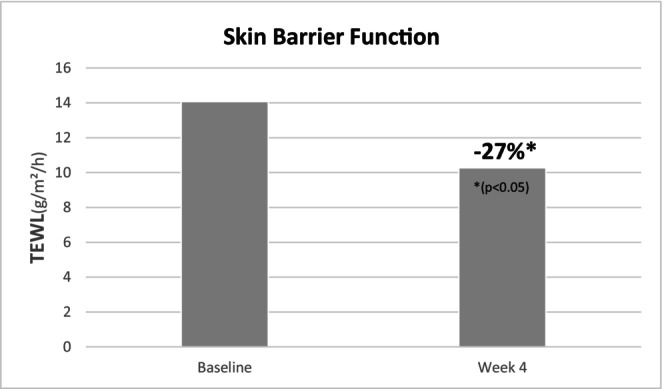
Evolution of TEWL over the 28‐day application period.

**FIGURE 3:**
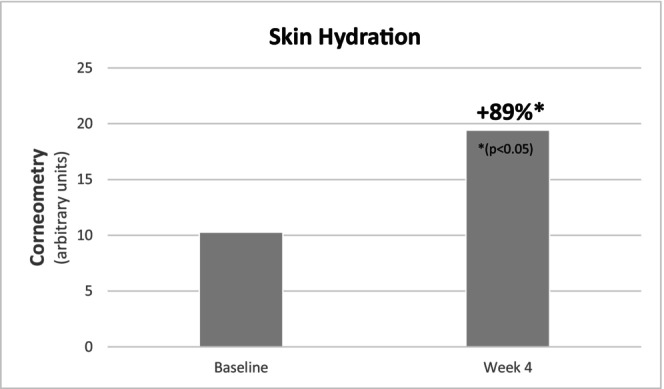
Evolution of skin hydration over the 28‐day application period.

Instrumental evaluations confirmed the clinical observations. TEWL, a marker of skin barrier integrity, decreased significantly by 27% over the 4‐week period (14.07 ± 1.54 to 10.27 ± 1.50 g/m^2^/h; *p* = 0.007), indicating enhanced barrier function (Figure 2). In parallel, skin hydration, measured via corneometry, showed a substantial increase of 89%, from 10.28 ± 1.57 to 19.42 ± 2.86 a.u. (*p* < 0.001) (Figure 3), providing evidence of a significant moisturizing effect of the regimen.

### 3.3 Quality of Life and Patient Well‐Being

**FIGURE 4:**
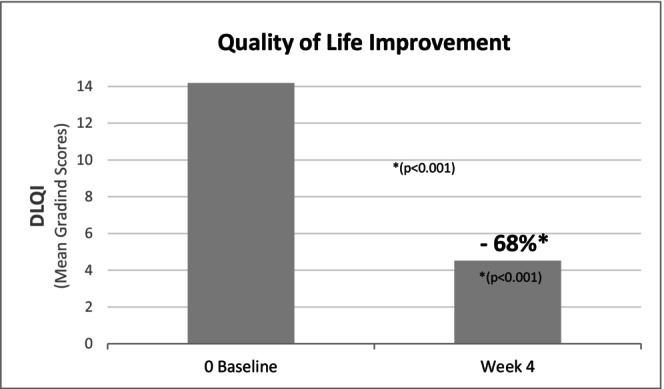
Evolution of quality of life (DLQI score) over the 28‐day application period.

Improvements in QoL were reflected by a significant reduction in the DLQI (Figure 4), which decreased from 14.19 ± 6.40 at D0 to 4.52 ± 4.18 at D28 (*p* < 0.001), representing a 68% improvement. Several individual DLQI items also demonstrated marked improvements. Notably, skin‐related perceived discomfort (itching, soreness, stinging) declined by 56% (*p* < 0.001), while emotional distress, assessed through embarrassment or self‐consciousness, was reduced by 60% (*p* < 0.001). Social and functional impairments were also alleviated: difficulties in social or leisure activities decreased by 86% (*p* < 0.001), interference with sport by 77% (*p* = 0.002), and problems with close personal relationships by 87% (*p* = 0.003). The impact on professional or academic activities also improved significantly by 78% (*p* = 0.039).

### 3.4 Perceived Efficacy and Cosmetic Acceptability

At D28, most participants rated the regimen highly in terms of both efficacy and cosmetic qualities. According to the efficacy questionnaire, 100% of participants reported a feeling of skin relief, and over 90% reported immediate and durable soothing, comfort, and moisturization. Perceptions of skin being nourished, protected, and strengthened were also reported in 90% of subjects. In particular, emotional well‐being appeared positively impacted, with 90% of subjects stating the regimen was a source of well‐being and 76% reporting improved sleep quality. The application was also described as pleasurable by 95% of subjects.

Ninety percent of patients rated the cosmetic acceptability of the products as “good” to “very good”. Similarly, 81% of subjects considered the efficacy of the regimen to be “good” to “very good”, supporting a high level of satisfaction and perceived benefit.

Moreover, in comparison to their usual skincare products, 71% of subjects reported that the investigational dermocosmetic regimen was more effective in improving the symptoms of AD, including dryness, itching, and irritation. None of the participants considered their usual products to be more effective than the tested regimen, highlighting a perceived added benefit of this dedicated routine over their standard skincare practices.

A total of 95% expressed willingness to continue using the products, and 86% would recommend this lipid‐replenishing regimen to others.

## 4 Discussion

This clinical study demonstrates that the application of a lipid‐replenishing dermocosmetic regimen, comprising a Cleansing Replenishing Oil and Emollient Balm, leads to significant improvements in both skin parameters and patient emotional well‐being in adults with moderate to severe AD.

Over a 4‐week period, SCORAD and TEWL decreased significantly, while skin hydration increased markedly. In parallel, strong decreases in pruritus and insomnia were reported, and a 68% improvement in QoL was assessed by the DLQI. These results highlight the multidimensional benefits of the dermocosmetic regimen that addresses not only cutaneous manifestations but also the psychosocial burden of AD.

The clinical and instrumental improvements may be mechanistically linked to the specific composition of the products. The emollient balm, enriched with shea butter, linseed oil, and beeswax, provides essential lipids that restore the hydrolipidic film and supply omega fatty acids, known to reduce inflammation and promote epidermal repair. Ex vivo studies have further shown that topical application of the DA PROTECT Emollient Balm can upregulate filaggrin, a key protein for keratinocyte differentiation and the generation of natural moisturizing factors, thereby directly supporting barrier recovery [10, 11].

In addition to emollients, the role of hygiene is increasingly recognized as a key element in AD management, particularly when cleansing products are designed to strengthen the skin barrier. The cleansing oil, formulated with linseed and camelina oil extracts, ensures gentle hygiene while delivering barrier‐supportive lipids. Unlike conventional cleansers, which may disrupt the stratum corneum, emollient‐rich cleansing oils maintain hydration and initiate barrier protection from the first step of the skincare routine [12], when the skin is most vulnerable to disruption.

Together, this combination offers a synergistic approach: the cleansing oil prevents barrier damage during washing, while the balm promotes lipid replenishment and barrier repair. This dual action explains the consistent improvements observed across both clinical symptoms and patient‐reported outcomes.

Beyond the topical benefits, the regimen alleviated AD‐related psychosocial burden, as reflected by better sleep and QoL scores. The significant reduction in pruritus and insomnia—two symptoms most directly associated with patient distress—demonstrates the capacity of dermocosmetic care to extend benefits beyond the skin, contributing to emotional and functional recovery. These improvements are consistent with findings from recent controlled studies [13], which reported that emollient regimens led to better patient adherence and reduced corticosteroid use, likely due to enhanced tolerability and sensorial acceptance.

Patient‐reported satisfaction was notably high, with over 90% describing the regimen as comforting and effective, and 95% indicating their intention to continue use. The absence of adverse events and strong cosmetic acceptability further support the regimen's suitability for long‐term maintenance, especially in sensitive and diverse skin types. Taken together, these outcomes support the hypothesis that integrating lipid‐replenishing cleansing with intensive moisturization represents a comprehensive dermocosmetic approach, capable of addressing the multidimensional needs of patients with moderate to severe AD.

Furthermore, emerging insights from neuroscience may help deepen our understanding of how dermocosmetic routines impact patient emotional well‐being. Beyond observable improvements in pruritus or sleep, neurocognitive and sensorial approaches could provide objective data on how skincare influences emotional state, stress modulation, and overall QoL. Such research may contribute to a broader definition of “feel‐good dermatology,” bridging the gap between skin well‐being and mental well‐being.

This study has several limitations. The open‐label design and relatively small sample size may restrict the generalizability of the results. Moreover, the absence of pathological and molecular investigations and the lack of long‐term follow‐up limit the mechanistic understanding and the durability assessment of the observed benefits. Nonetheless, the consistent improvements across both instrumental and patient‐reported outcomes reinforce the clinical relevance of the findings. Future randomized controlled trials with larger and more diverse cohorts, integrating molecular endpoints and extended follow‐up, are warranted to confirm and expand these results.

## 5 Conclusion

In conclusion, the results of this study demonstrate that a lipid‐replenishing dermocosmetic regimen comprising a cleansing oil and emollient balm significantly enhances both the clinical and psychosocial outcomes for patients suffering from moderate to severe AD. Over a 4‐week period, this regimen successfully improved skin barrier function, reduced pruritus and sleep disturbances, and led to substantial improvements in overall QoL as evidenced by the DLQI. The substantial reduction in TEWL, coupled with a marked increase in skin hydration, underscores the effectiveness of this approach in supporting long‐term skin health and comfort. Importantly, patient satisfaction and cosmetic acceptability were high, with participants reporting immediate and lasting relief, and most expressing a strong willingness to continue the use of the products. These findings highlight the dual role of dermocosmetic care not only in addressing the cutaneous symptoms of AD but also in improving emotional well‐being, thereby emphasizing the critical role of comprehensive skincare routines in a patient‐centered management of AD. Future research with larger and more diverse populations is necessary to confirm the long‐term benefits and explore the broader implications of such dermocosmetic interventions in the clinical management of AD.

## Author Contributions

Both authors contributed equally to the study conception, clinical study oversight, and manuscript preparation. They took part in drafting, revising, and critically reviewing the article; gave final approval of the version to be published; agreed on the journal to which the article has been submitted; and accept accountability for all aspects of the work.

## Funding

The authors have nothing to report.

## Ethics Statement

This study was conducted in accordance with the ethical principles outlined in the Declaration of Helsinki (1964) and its most recent amendments. The protocol complied with all applicable ethical standards, with the exception of Principle No. 35 concerning registration in a publicly accessible database.

## Consent

All participants provided written informed consent prior to inclusion in the study.

## Conflicts of Interest

Marie Gaudin and Aurélie Geoffroy are employees of Laboratoires NIGY.

## Data Availability

The datasets generated and analyzed during the current study are available from the corresponding author on reasonable request.
